# Stress Urinary Incontinence post-Holmium Laser Enucleation of the Prostate: a Single-Surgeon Experience

**DOI:** 10.1590/S1677-5538.IBJU.2019.0411

**Published:** 2020-03-25

**Authors:** Akhil K. Das, Seth Teplitsky, Thenappan Chandrasekar, Tomy Perez, Jenny Guo, Joon Yau Leong, Patrick J. Shenot

**Affiliations:** 1 Department of Urology Sidney Kimmel Medical College Thomas Jefferson University PhiladelphiaPA USA Department of Urology, Sidney Kimmel Medical College at Thomas Jefferson University, Philadelphia, PA, USA

**Keywords:** Epidemiology, physiopathology [Subheading], Urinary Incontinence

## Abstract

**Purpose:**

To identify incidence and predictors of stress urinary incontinence (SUI) following Holmium laser enucleation of the prostate (HoLEP).

**Materials and Methods:**

We performed a retrospective review of 589 HoLEP patients from 2012-2018. Patients were assessed at pre-operative and post-operative visits. Univariate and multivariate regression analyses were performed to identify predictors of SUI.

**Results:**

52/589 patients (8.8%) developed transient SUI, while 9/589 (1.5%) developed long-term SUI. tSUI resolved for 46 patients (88.5%) within the first six weeks and in 6 patients (11.5%) between 6 weeks to 3 months. Long-term SUI patients required intervention, achieving continence at 16.4 months on average, 44 men (70.9%) with incontinence were catheter dependent preoperatively. Mean prostatic volume was 148.7mL in tSUI patients, 111.6mL in long-term SUI, and 87.9mL in others (p <0.0001). On univariate analysis, laser energy used (p <0.0001), laser “on” time (p=0.0204), resected prostate weight (p <0.0001), overall International Prostate Symptom Score (IPSS) (p=0.0005), and IPSS QOL (p=0.02) were associated with SUI. On multivariate analysis, resected prostate weight was predictive of any SUI and tSUI, with no risk factors identified for long-term SUI.

**Conclusion:**

Post-HoLEP SUI occurs in ~10% of patients, with 1.5% continuing beyond six months. Most patients with tSUI recover within the first six weeks. Prostate size >100g and catheter dependency are associated with increased risk tSUI. Larger prostate volume is an independent predictor of any SUI, and tSUI.

## INTRODUCTION

Benign prostatic hyperplasia (BPH) is a common condition affecting many older men. In the United States, more than 70% of men aged 60-69 years have symptoms associated with BPH. Currently, lower urinary tract symptoms (LUTS) impact almost 80% of men older than 70 ( 1 ).

Historically, transurethral resection of the prostate (TURP) has been the gold standard for endoscopic management of BPH ( 2 ). This technique, although effective, has many potential adverse effects and limitations which have prompted the advent of newer treatment modalities for BPH ( 3 - 5 ). Holmium-laser enucleation of the prostate (HoLEP) is one of the most prominent newer modalities. HoLEP is size independent and can be used for enucleation of prostates over 100g, which has traditionally been a limitation of TURP. Recent studies have shown that HoLEP is equally as effective, and potentially more effective, when compared to TURP and open simple prostatectomy across a variety of outcomes ( 4 ).

With HoLEP now recognized in the AUA guidelines as a viable alternative treatment option for those with moderate to severe LUTS, it is important to understand better the adverse event profile associated with this procedure ( 6 ). In our institutional experience, the most common complication encountered with HoLEP is postoperative stress urinary incontinence (SUI). The vast majority of SUI seen after HoLEP is transient, with the majority of cases resolving within one year ( 7 ). We have found that transient SUI (tSUI) represents one of the most common complaints affecting patient satisfaction and the quality of life postoperatively. Recent reports note that the rates of postoperative SUI range between 1.4% and 44% following HoLEP ( 8 - 11 ). The wide range in reported SUI rates is most likely multifactorial and may be due to different operative techniques, surgeon experience, and patient-specific factors. Unfortunately, many of these studies are limited by small sample sizes and technique heterogeneity.

With this study, we aim to more accurately define SUI rates using a large single-surgeon single-institutional experience and to identify the incidence and predictors of SUI following HoLEP. With this knowledge, surgeons can better counsel patients regarding the procedure allowing for more informed patient decision making and improve patient satisfaction.

## MATERIALS AND METHODS

We performed an IRB approved (Control #12D.50) retrospective chart review of all patients undergoing HoLEP at our institution between January 2012 and June 2018. Our review included the charts of all patient who underwent HoLEP at our institution within this time period under the care of a single surgeon. The exclusion criteria for this review include incomplete surgical resection and lack of post-operative follow-up. Baseline demographic data collected included age, body mass index (BMI), serum prostate-specific antigen (PSA), prostate volume, peak Uroflow rate, mean Uroflow rate, PVR volume, IPSS score, and IPSS QOL rating. All patients underwent urodynamic testing (Laborie Medical Technologies®) before undergoing surgery to confirm the diagnosis of bladder outlet obstruction. All procedures were performed by a single experienced surgeon (A.D.) who had performed more than one thousand HoLEP cases before the study period. Postoperative clinic visits were conducted within two weeks, at six weeks, and at three months. Assessment at postoperative visits included the IPSS questionnaire, PVR, and Uroflow testing.

Our trilobar HoLEP technique has been described previously, but in brief, a 26 French (Fr) continuous flow resectoscope with a laser bridge adapter and an endoscopic camera are utilized ( 12 ). The laser fiber is passed through a 6Fr open-ended ureteral catheter. A 100 Watt holmium laser with an end-firing 550-micron laser fiber is used with energy settings of 2.0J and 50Hz. After trilobar enucleation is completed, a morcellator, grasper, or both are used to clear the bladder of any prostatic tissue.

At postoperative visits, SUI was assessed clinically, which was defined as incontinence during activity with a patient-reported negative impact on quality of life. Any patient who reported incontinence which necessitated the utilization of undergarment pads or diapers was considered “incontinence”, while those who did not report needing any pads or diapers were considered continent for our study purposes. SUI was differentiated from other forms of incontinence by careful history taking and physical examination in the clinic. Patients with different types of incontinence, such as urge or mixed, were excluded from this study. For study purposes, SUI was considered transient if it resolved within six months of the procedure date, in following with previous literature ( 6 ). Any leakage beyond six months was deemed to be long-term SUI.

Additional risk factors assessed included prostate size as measured by TRUS, CT, or MRI imaging. Patients were risk stratified based on preoperative prostate size (>100g or ≤100g) and pre-operative catheter dependency status (clean intermittent catheterization and continuous urethral drainage).

Univariate analysis for baseline demographics and perioperative risk factors were completed using the chi-square test for categorical variables and ANOVA for comparison of continuous variables. Multivariable logistic regression was completed to identify factors predictive of increased risk for any SUI, transient SUI, and long-term SUI after HoLEP. Significant factors from the univariate analysis were included in the multivariable analysis. Analyses were completed using SPSS®, version 23.0.

## RESULTS

Five hundred eighty nine men undergoing HoLEP during the study period were identified. Postoperative tSUI occurred in 52 men (8.8%), while 9 (1.5%) had long-term SUI, for a total of 61 (10.4%) patients who experienced any SUI after HoLEP. Of the patients who experienced tSUI, all had their incontinence resolved within three months. 46 men (88.5%) with tSUI had full resolution of incontinence within the first six weeks, while the remaining 6 men (11.5%) resolved between six weeks and three months.

Table-1 highlights preoperative baseline characteristics as well as perioperative results. Except for pre-operative prostate size (tSUI: 148.7±56.8mL, long-term SUI: 98.0±50.1mL, no SUI: 92.2±50.6mL, p <0.0001), pre-operative Qmax (tSUI: 14.7±23.5mL/s, long-term SUI: 11.5±10.8mL/s, no SUI: 8.6±9.9mL/s, p=0.0009) and pre-operative catheter dependence (tSUI: 71.2% vs. long-term SUI: 77.8% vs. no SUI: 38.1%, p <0.0001), there was no significant difference between men who developed transient SUI, long-term SUI, and those who did not.

**Table 1:** Baseline Characteristics, Preoperative, and Perioperative Data.

	Patients with no SUI (n=528)	tSUI patients (n=52)	Long-term SUI (n=9)	p-value
**Preoperative Data**				
Age	70.6±8.5	72.0±8.9	65.6±5.7	0.1027
BMI	28.7±7.8	29.6±5.8	28.2±4.1	0.7026
Serum PSA (ng/mL)	10.03±47.45	6.7±7.4	9.0±7.1	0.9388
Prostate Size (mL)	92.2±50.6	148.7±56.8	111.6±48.5	<0.0001
Pre-Op Uroflow Peak Flow (mL/s)	8.6±9.9	14.7±23.5	11.5±10.8	0.0009
Pre-Op Uroflow Mean Flow (mL/s)	3.4±2.5	3.7±3.2	3.5±1.8	0.9367
Pre-Op Post Void Residual (mL)	238.8±249.2	297.0±298.1	185.2±112.3	0.3030
Pre-Op IPSS Results	19.7±8.5	17.9±9.8	19.4±5.8	0.5897
Pre-Op IPSS QOL Results	3.6±1.2	3.5±1.5	3.8±0.7	0.8231
Pre-operative Catheterization (N, %)	201 (38.1%)	37 (71.2%)	7 (77.8%)	<0.0001
**Perioperative Data**				
Laser Energy Used (kJ)	339.3±190.4	514.4±151.4	434.3±145.8	<0.0001
Laser On Time (min)	118.7±72.8	163.5±89.7	174.2±67.4	0.0204
Resected Prostate Weight (g)	70.2±42.8	135.5±70.5	103.2±52.4	<0.0001
Post-Op Catheterization Time (days)	5.5±3.5	4.9±2.1	6.6±4.6	0.6139
Post-Op Uroflow Peak Flow (mL/s)	24.3±17.6	24.0±11.6	24.8±9.5	0.9926
Post-Op Uroflow Mean Flow (mL/s)	6.4±4.8	5.1±3.2	6.4±1.7	0.6384
Post-Op Post Void Residual (mL)	63.4±89.3	63.4±78.5	10.3±10.5	0.3414
Post-Op IPSS Results	6.8±5.9	9.5±8.3	16.7±11.4	0.0005
Post-Op IPSS QOL Results	1.1±1.4	1.8±1.4	2.4±2.0	0.0214

Continuous data is presented as mean ± standard deviation; categorical data as proportions

With regard to perioperative and postoperative results, patients who developed SUI were found to have greater laser energy used (tSUI: 514.4±151.4 kJ vs. long-term SUI: 434.3±145.8 kJ vs. no SUI :339.3±190.4 kJ, p <0.0001), longer laser “on” time (tSUI: 163.5±89.7 min, long-term SUI: 174.2±67.4 min, no SUI: 118.7±72.8 min, p=0.0204), larger resected prostate weight (tSUI: 135.5±70.5g, long-term SUI: 103.2±52.4g, no SUI: 70.2±42.8g, p <0.0001), higher overall IPSS score (tSUI: 9.5±8.3, long term SUI: 16.7±11.4, no SUI: 6.8±5.9, p=0.0005) and IPSS QOL scores (tSUI: 1.8±1.4, long term SUI: 2.4±2.0, no SUI: 1.1±1.4, p=0.0214).

When patients with tSUI were stratified by preoperative prostate volume of >100g (n=44) or ≤100g (n=8), 8 men (100%) with prostates ≤100g had the resolution of tSUI within 6 weeks. In the 44 men with larger (>100g) prostates, 38 (86.3%) had the resolution of tSUI within 6 weeks, while the remaining 6 (13.6%) had the resolution between 6 weeks to 3 months. There was not a statistical significance (p=0.2394) in recovery time when comparing larger prostates (>100g) to smaller prostates (≤100g).

On multivariable logistic regression analysis, we performed multiple analyses looking for predictive factors predisposing patients to any SUI ( Supplementary Table-1.1 ), tSUI ( >Supplementary Table-1.2 ), and long-term SUI ( Supplementary Table-1.3 ). Results showed that only resected prostate weight was a significant predictor of developing any SUI (HR 1.020, 95% CI 1.007-1.033, p <0.05) and tSUI (HR 1.019, 95% CI 1.006-1.032, p <0.05). There were no risk factors identified for long-term SUI patients.

## DISCUSSION

HoLEP has struggled to gain widespread adoption within the urology community due to a well-established steep learning curve, requiring up to 50 cases to become proficient ( 11 , 13 , 14 ) The potential development of tSUI has also been a limiting factor in its uptake. SUI, the involuntary leakage of urine, is distressing and has been shown to decrease the quality of life in patients ( 15 ). Patient distress likely plays a role in the avoidance of this prostate reducing technique by surgeons.

In our study, we evaluated the incidence and predictors of SUI after HoLEP. We then looked to compare our results to those of other prostate reducing procedures in the literature. In patients undergoing TURP, the incidence of long-term stress incontinence is rare (~1%), although 30-40% of patients have tSUI resolving within six months ( 16 , 17 ). On the other hand, in men undergoing open simple prostatectomy (OP), there is a much higher incidence of long-term incontinence ranging between 1-40% depending on the technique utilized, with more current data showing 20.2% of patients becoming permanently incontinent ( 18 - 21 ). Further studies have shown a 38.6% incidence of tSUI in the three months following simple prostatectomy ( 22 ). In comparison, the incidence of long-term and transient SUI in our single-surgeon series is 1.5% and 8.8%, respectively. Our results suggest improved surgical outcomes compared to the published literature.

Both HoLEP and simple prostatectomy seek to provide complete enucleation of the adenoma. Our HoLEP technique involves endoscopic dissection of the adenoma in a retrograde fashion, from the distal to the proximal attachments. HoLEP allows for distal visualization of the adenoma, compared to the blind approach taken with OP. We feel that this visualization confers an advantage, as visual landmarks allow for the surgeon to avoid damage to the sphincter, and may play a role in lower incontinence rates seen in HoLEP versus OP.

Variable tSUI rates after HoLEP have been reported in the literature. Previous studies have outlined this complication as occurring in anywhere from 1.4-44% of patients, of which the vast majority recover full continence by one year ( 10 , 11 , 23 ). A more recent large cohort study from Japan reported a tSUI rate after HoLEP of 16.6%, which is more consistent with our series ( 8 ). However, while the extreme variation in reported SUI rates postoperatively is likely a function of small sample sizes, variable operative experience, heterogeneous operative techniques, and possible prior bladder dysfunction, our series has the advantage of being a large single-surgeon series. Shah et al. assessed the amount of time required to regain bladder control following HoLEP and found that it took 42.3 days ( 24 ). These results are consistent with our study, in which all patients who developed tSUI had complete a recovery by three months. We assessed if a larger prostate size was associated with rate of tSUI recovery. All six patients who took over 6 weeks to recover had large prostate volume, while no patients took over 6 weeks with a low prostate volume. Though the analysis did not show statistical significance (p=0.2394), the absolute numbers suggest that men with larger prostate may have a slightly slower rate of recovery.

This study also sought to uncover risk factors that may predispose patients to SUI, both transient and long-term. There was a statistical difference seen amongst the three groups on analysis for preoperative prostate size, preoperative Qmax, laser “on” time, laser energy used, and resected prostate volume. A novel finding of our study was the association of preoperative catheter dependence with postoperative incontinence. The cause of this correlation remains unknown, but it is possible that patients requiring preoperative catheterization have more severe BPH, and therefore require longer endoscopic manipulation, which predisposes the patients to SUI. The only prior study looking at risk factors by Nam et al. identified increasing age and operative time as risk factors ( 25 ). However, on our multivariate logistic regression analysis, only resected prostate weight was a significant predictor of developing any SUI (HR 1.020, 95% CI 1.007-1.033, p <0.05) and tSUI (HR 1.019, 95% CI 1.006-1.032, p <0.05).

Our study showed the rate of long-term incontinence to be very low. While no preoperative risk factors predisposing patients to long-term SUI were identified on multivariate regression, post-analysis chart review of these 9 patients uncovered a high rate of neurological comorbidities. Further investigation showed that 8/9 (88.9%) patients had a significant neurological history. However, as we cannot accurately capture the incidence of neurological comorbidities that exist within the rest of the population, a comparison was not possible. Of the 8, 7 had spinal pathology, including spinal stenosis and degenerative disc disease, while 1 had myasthenia gravis. Critically, all of these patients were able to achieve either complete resolution of SUI or reduction to less than 2 pads/day. The management strategies for these patients included initial pelvic floor exercise therapy, and in those still unsatisfied, coaptite injections. Multiple coaptite injections were given to patients who had partial responses. Only one patient went on to require AUS implantation. These 9 patients achieved a satisfactory level of continence at an average of 16.4 months after the completion of their HoLEP.

When assessing postoperative QOL outcomes, we found that men with tSUI and long-term SUI had worse postoperative IPSS scores (both worse subjective symptoms and quality of life responses) when compared to those patients who did not experience tSUI. As expected, SUI had a substantial negative impact on quality of life. This result is in line with previous reports showing the quality of life impact that incontinence can have on patients ( 26 ).

One possible cause of incontinence seen after surgery is sphincter dysfunction, which is likely the result of prolonged endoscopic manipulation. Endoscopic procedures are thought to cause trauma directly to the sphincter, leading to this dysfunction. This dysfunction is thought to be temporary, causing the transient nature of the SUI ( 8 , 11 , 27 ) A larger prostate size leads to longer operative times, which may explain the correlation between tSUI and both larger prostates and longer operative times. The longer laser time and laser energy used, as well as the heavier weight of resected prostate tissue, are all associated with a larger prostate. All of our results seem to indicate that larger prostates cause longer operations, and therefore increased endoscopic manipulation and risk for tSUI via sphincter dysfunction. As many of the variables associated with tSUI on univariate analysis were surrogate markers of prostate size, this may explain why they were not independently associated with tSUI on multivariate regression analysis.

This study is not devoid of limitations, including those inherent to a retrospective study. SUI characterization was primarily dependent on physician documentation. Missing data, especially from operative variables, also limited the robustness of the multivariable analysis. Comorbidities, such as diabetes, that may have been associated with SUI development were not adequately captured.

Regardless of these limitations, this study still represents the largest single-surgeon experience with HoLEP and provided valuable data regarding the incidence, time course and predictors of post-operative tSUI. Transient SUI after HoLEP has notable implications for patient quality of life, which may contribute to the hesitancy in the widespread adoption of HoLEP. Our study demonstrates this tSUI resolves in the majority of patients, usually within the first six weeks. Furthermore, by identifying risk factors that predispose patients to tSUI, preoperative counseling can be enhanced, thus mitigating possible patient frustration and improving both patient and physician satisfaction. This may also provide data for better patient selection in order to avoid these complications after the HoLEP procedure. We feel this study shows the need for future prospective trials and further inquiry into ways to prevent this complication.

## CONCLUSIONS

Overall, our study helps to highlight the relatively low rate of incontinence (10.4%) seen after HoLEP from an experienced surgeon, with 8.8% being transient and 1.5% being long-term. Our results suggest that the overwhelming majority of patients with tSUI recover within the first 6 weeks following HOLEP. Prostate size greater than 100g and catheter dependency are associated with increased risk of developing tSUI, and larger prostate volume is an independent predictor of tSUI. While long-term SUI was rare (1.5%), it is possible that neurologic complications may be a contributing factor, though this requires further study. Those with long-term SUI are most often able to achieve continence with further intervention.

## Supplementary

**Table:** Supplementary Table 1.1 - Results from multivariable logistic regression analysis assessing for risk factors for any SUI in HoLEP patients.

Variables	OR (95% CI)	p
Age (years)	0.981 (0.928-1.037)	0.494
BMI (Kg/m^2^)	0.983 (0.923-1.047)	0.597
Prostate Volume (mL)	1.009 (0.997-1.021)	0.133
Laser Energy Used (Kj)	0.990 (0.996-1.003)	0.763
Laser On Time (min)	0.997 (0.990-1.005)	0.463
Resected Prostate Volume (cc)	1.020 (1.007-1.033)	0.002

**Table:** Supplementary Table 1.2 - Results from multivariable logistic regression analysis assessing for risk factors for tSUI in HoLEP patients.

Variables	OR (95% CI)	p
Age (years)	1.013 (0.949-1.083)	0.694
BMI (Kg/m^2^)	0.987 (0.925-1.054)	0.697
Prostate Volume (mL)	1.011 (0.997-1.025)	0.113
Laser Energy Used (Kj)	1.001 (0.996-1.006)	0.731
Laser On Time (min)	0.995 (0.986-1.004)	0.259
Resected Prostate Volume (cc)	1.019 (1.006-1.032)	0.004

**Table:** Supplementary Table 1.3 - Results from multivariable logistic regression analysis assessing for risk factors for long-term SUI in HoLEP patients.

Variables	OR (95% CI)	p
Age (years)	0.915 (0.825-1.014)	0.090
BMI (Kg/m^2^)	0.964 (0.830-1.120)	0.630
Prostate Volume (mL)	1.002 (0.978-1.026)	0.896
Laser Energy Used (Kj)	0.997 (0.990-1.003)	0.345
Laser On Time (min)	1.006 (0.994-1.019)	0.308
Resected Prostate Volume (cc)	1.009 (0.985-1.034)	0.456

## ABBREVIATIONS

SUI: Stress urinary incontinence
tSUI: Transient stress urinary incontinence
HoLEP: Holmium laser enucleation of prostate
BPH: Benign prostatic hyperplasia
LUTS: Lower urinary tract symptoms
TURP: Transurethral resection of prostate
PVR: Post-void residual
OP: Open prostatectomy
Qmax: Peak flow
IPSSx: International prostate symptom score
QOL: Quality of life
PSA: Prostate-specific antigen
Fr: French
